# Metabolite and transcriptome analyses reveal the effects of salinity stress on the biosynthesis of proanthocyanidins and anthocyanins in grape suspension cells

**DOI:** 10.3389/fpls.2024.1351008

**Published:** 2024-03-21

**Authors:** Kainan Zhao, Yibin Lan, Ying Shi, Changqing Duan, Keji Yu

**Affiliations:** ^1^ Center for Viticulture and Enology, College of Food Science and Nutritional Engineering, China Agricultural University, Beijing, China; ^2^ Key Laboratory of Viticulture and Enology, Ministry of Agriculture and Rural Affairs, Beijing, China; ^3^ College of Biological Sciences and Biotechnology, Beijing Forestry University, Beijing, China

**Keywords:** grape, proanthocyanidins, anthocyanins, salinity, suspension cells, flavonoid biosynthesis, transcription factors, WGCNA

## Abstract

Proanthocyanidins (PAs) and anthocyanins are flavonoids that contribute to the quality and health benefits of grapes and wine. Salinity affects their biosynthesis, but the underlying mechanism is still unclear. We studied the effects of NaCl stress on PA and anthocyanin biosynthesis in grape suspension cells derived from berry skins of *Vitis vinifera* L. Cabernet Sauvignon using metabolite profiling and transcriptome analysis. We treated the cells with low (75 mM NaCl) and high (150 mM NaCl) salinity for 4 and 7 days. High salinity inhibited cell growth and enhanced PA and anthocyanin accumulation more than low salinity. The salinity-induced PAs and anthocyanins lacked C5’-hydroxylation modification, suggesting the biological significance of delphinidin- and epigallocatechin-derivatives in coping with stress. The genes up-regulated by salinity stress indicated that the anthocyanin pathway was more sensitive to salt concentration than the PA pathway, and WGCNA analysis revealed the coordination between flavonoid biosynthesis and cell wall metabolism under salinity stress. We identified transcription factors potentially involved in regulating NaCl dose- and time-dependent PA and anthocyanin accumulation, showing the dynamic remodeling of flavonoid regulation network under different salinity levels and durations. Our study provides new insights into regulator candidates for tailoring flavonoid composition and molecular indicators of salt stress in grape cells.

## 1 Introduction

Grapevine (*Vitis vinifera* L.) is one of the most economically important fruit crops in the world. It produces a wide range of flavonoids, especially in the berry skins of red varieties. Among flavonoids, proanthocyanidins (PAs) and anthocyanins are important compounds that contribute to the quality and health benefits of grapes and wine (Garrido-Bañuelos et al., 2022). PAs, also known as condensed tannins, are polymers of flavan-3-ols that can affect the astringency, bitterness, and mouthfeel of wine (Ma et al., 2014). Anthocyanins are glycosylated derivatives of anthocyanidins that are responsible for the red or purple colors of grape skins and red wine (Zhang et al., 2022).

The biosynthesis of anthocyanins and PAs in plants starts from the common phenolic precursor, phenylalanine, which is catalyzed to cinnamic acid by phenylalanine ammonia-lyase (PAL) (Rasmussen and Dixon, 1999). Cinnamic acid is sequentially catalyzed by cinnamate 4-hydroxylase (C4H) and 4-coumarate-CoA ligase (4CL) to form 4-coumaroyl-CoA (Schilmiller et al., 2009). Chalcone synthase (CHS) catalyzes the condensation of 4-coumaroyl-CoA with three molecules of malonyl-CoA to produce naringenin chalcone, the first committed intermediate of the flavonoid pathway (Hrazdina et al., 1986). Naringenin chalcone is further isomerized to naringenin by chalcone isomerase (CHI) (Moustafa and Wong, 1967), followed by hydroxylation mediated by various cytochrome P450 enzymes, such as flavanone 3-hydroxylase (F3H), flavonoid 3’-hydroxylase (F3’H), and flavonoid 3’,5’-hydroxylase (F3’5’H) to produce dihydroflavonols (Forkmann et al., 1980; Britsch and Grisebach, 1986; Menting et al., 1994). Dihydroflavonols are subsequently reduced by dihydroflavonol 4-reductase (DFR) to form 2,3-*trans*-leucoanthocyanidins (Fischer et al., 1988). These 2,3-*trans*-leucoanthocyanidins can undergo conversion into anthocyanidins by anthocyanidin synthase (ANS) with the aid of glutathione transferase (GST) or into 2,3-*trans*-flavan-3-ols (e.g., (+)-catechin) by leucoanthocyanidin reductase (LAR) (Yu et al., 2019; Eichenberger et al., 2023). Anthocyanidins are then glycosylated by UDP-glucose:flavonoid 3-*O*-glucosyltransferase (UFGT) to form anthocyanins (Ford et al., 1998), which can be further modified by acylation and methylation (Hugueney et al., 2009). At the downstream of ANS, anthocyanin reductase (ANR) utilizes ANS product intermediates to produce 2,3-*cis*-flavan-3-ols (e.g., (-)-epicatechin) and 2,3-*cis*-leucoanthocyanidins (Jun et al., 2021). The polymerization of PAs is believed to be non-enzymatic, which requires flavan-3-ols as starter units, and leucoanthocyanidins or their conjugates derived carbocation as the extension units (Jun et al., 2021; Yu et al., 2022, 2023).

A number of transcription factors that modulate the expression of flavonoid pathway genes have been identified in the context of berry development. Deluc et al. (2006, 2008) demonstrated that VvMYB5a and VvMYB5b activate the upstream flavonoid pathway to enhance both PA and anthocyanin production. Subsequently, VvMYBPA1, VvMYBPA2, and VvMYBPAR were identified as the three transcriptional activators that specifically target the PA branch genes *VvLAR1* and *VvANR* (Bogs et al., 2007; Terrier et al., 2009; Koyama et al., 2014). VvMYBC2-L1, VvMYBC2-L2, and VvMYBC2-L3 are negative regulators of PA biosynthesis that downregulate *VvMYBPA1*, *VvMYBPA2*, and several PA structural genes, including *VvLAR*s and *VvANR* (Huang et al., 2014; Cavallini et al., 2015; Zhu et al., 2019). Our previous studies showed that VvMYB86 promotes PA accumulation by tuning the flux between PA and anthocyanin pathways (Cheng et al., 2021), while VvbHLH93 inhibits PA production by broadly targeting flavonoid pathway genes (Cheng et al., 2022). Confirmed by transgenic grape suspension cells, VvNAC17 is an activator of both PA and anthocyanin pathways (Badim et al., 2022). Recently, Wei et al. (2023) found that VvWRKY70 is a bi-functional transcription factor that simultaneously represses norisoprenoid and flavonoid biosynthesis.

Salinity stress is a predominant issue for agriculture in arid and semi-arid regions, and has the potential to impair grape yield and wine quality (Zhou‐Tsang et al., 2021). Plant responses to salinity stress involve a biphasic mechanism, which includes an early osmotic phase and a late ionic phase (Munns and Tester, 2008), both of which can lead to excessive production of reactive oxygen species (ROS) (Chele et al., 2021). The accumulation of flavonoids under salinity stress is considered to be a strategy of antioxidation or ROS scavenging in plants (Shah and Smith, 2020). However, most studies have focused on the effect of salinity stress on anthocyanin biosynthesis, while the impact on PA accumulation has been largely neglected. In model plant species, salinity stress has been shown to increase the accumulation of anthocyanins (Lotkowska et al., 2015; Kim et al., 2022), but the evidence for the effect of salinity on anthocyanin biosynthesis in grape berries is not consistent. The canopy NaCl spray experiments showed that moderate salinity (20 mM and 60 mM) and high salinity (100 mM and 150 mM) slightly enhanced and inhibited the biosynthesis of anthocyanin in grape berries, respectively (Li et al., 2013; Han et al., 2024). However, the irrigation with mild saline in the vineyard did not cause a significant change in the color density of wine products (Walker et al., 2000), suggesting that the effect of moderate salinity on anthocyanin accumulation is limited. The discrepancy may result from different grape varieties, various treatment methods, the choice of treatment time points, and the interaction between salinity and other environmental factors (Jan et al., 2021; Han et al., 2024). In addition, more experimental evidence is needed to elucidate how high salinity affects anthocyanin and PA biosynthesis in grapes.

Plant cell culture is a convenient system to study metabolite biosynthesis, cell development, and response to specific stimuli, as it allows for more precise control of treatment conditions (Onofrio et al., 2009; Almagro et al., 2014; Ayenew et al., 2015; Ric-Varas et al., 2020). Previous studies have shown that sugar, light, heat, and jasmonate can induce the biosynthesis of anthocyanins or PAs in grape cell cultures (Larronde et al., 1998; Vitrac et al., 2000; Decendit et al., 2002; Zhang et al., 2002; Onofrio et al., 2009; Ayenew et al., 2015). However, the effect of salinity on PA and anthocyanin metabolism in grape cells remains unclear. In this study, we explored the responses of PA and anthocyanin accumulation to different combinations of NaCl (75 mM and 150 mM) and treatment time (4 day and 7 day) in grape suspension cells. We found that the anthocyanin pathway was more sensitive to salt concentration than the PA pathway, and that flavonoid accumulation was coordinated with cell wall metabolism. We also identified transcription factors that potentially modulated the flavonoid regulation network in response to different salinity levels and durations. Our study reveals new flavonoid regulators and markers of salt stress in grape cells.

## 2 Materials and methods

### 2.1 Cell cultures and salinity treatment

The grape suspension cell system was established from *Vitis vinifera* L. Cabernet Sauvignon berry skin-derived callus described in our previous study (Cheng et al., 2022). The grape cell suspensions were cultivated according to the method of Osuga et al. (1999) with slight modifications. Briefly, the calli were transferred to 250 mL flask containing 60 mL of B5 basic liquid medium (3.21 g L^-1^, pH 5.8 to 6.0), supplemented with 20 g L^-1^ of sucrose, 2.5 g L^-1^ of acid-hydrolyzed casein, 0.2 mg L^-1^ of kinetin (KT), and 0.1 mg L^-1^ of naphthaleneacetic acid (NAA). The suspension cells were maintained at 25 ± 1°C in the dark with the a shaker speed of 95 rpm. Subcultures were performed every 14 days at a 1:2 ratio (v/v). For salinity treatment, NaCl was added to the fresh maintaining medium at the indicated concentration. Suspension cells were subjected to different NaCl concentrations (0 mM, 75 mM, and 150 mM) in 250 mL flasks, designated as the control group (Ctrl), low salinity group (L), and high salinity group (H), respectively. Each treatment was performed in three biological replicates. As the activation of anthocyanin pathway in grapevine is light-dependent, the experimental suspension cells were cultivated under the same condition as above, except for a 16 h/8 h light/dark cycle with a light intensity of 125 μmol m^-2^ s^-1^. Cells were harvested at the specified time intervals by vacuum filtration, washed with cold distilled water, and weighed. The samples for metabolite and gene expression analysis were frozen in liquid nitrogen and stored at -80°C until use.

### 2.2 Quantification of soluble and insoluble PAs

Two mL of suspension cell cultures were subjected to gravity sedimentation for 10 min, and the supernatant was discarded. The cell pellets were extracted with 1 mL of 70% (v/v) acetone/water containing 0.5% (v/v) ascorbic acid by vortexing for 30 min. The resulting mixture was centrifuged at 8,000*g* for 5 min, and the supernatant was collected. The extraction was repeated twice, and the combined supernatants were evaporated by nitrogen flow. The dry extracts were re-dissolved in 400 μL of 50% (v/v) methanol/water to obtain soluble PAs. The remained cell pellets after soluble PAs extraction contained insoluble PAs. Soluble and insoluble PAs were quantified using DMACA method and Butanol-HCl lysis assay, respectively, as described in our previous study (Cheng et al., 2022).

### 2.3 Quantification of total anthocyanin

To extract total anthocyanins from two mL of suspension cell cultures, the medium was removed and the cells were sonicated in 1 mL of 50% (v/v) methanol/water with an ice water bath for 20 min. The resulting homogenate was centrifuged at 8,000*g* for 5 min, and the supernatant was evaporated under the nitrogen flow. The dry extracts were re-dissolved in 400 μL of 50% (v/v) methanol/water. The quantification of total anthocyanins followed the pH-differential method optimized for grape cell cultures (Cheng et al., 2021).

### 2.4 PAs and anthocyanin profiling using LC/MS

To analyze the composition of PAs and anthocyanins, suspension cells were ground under liquid nitrogen and freeze-dried. For the cleavage of both soluble and insoluble PAs, 30 mg of dry powder was suspended in phloroglucinolysis buffer (0.5% (w/v) ascorbic acid, 0.3 N HCl, and 50 g/L phloroglucinol in methanol) and incubated at 50°C for 20 min. The reaction was stopped by adding 200 mM sodium acetate. After centrifugation at 8,000*g* for 5 min, the supernatant was evaporated under the nitrogen flow and re-dissolved in 400 μL of 50% (v/v) methanol/water. For the extraction of anthocyanins, 30 mg of dry powder was sonicated in 1 mL of 50% (v/v) methanol/water with an ice water bath for 20 min. The mixture was then centrifuged at 8,000*g* for 5 min and the supernatant was evaporated under the nitrogen flow and re-dissolved in 200 μL of 50% (v/v) methanol/water. Both PA building blocks and anthocyanins were detected using an Agilent 1200 HPLC system coupled with an Agilent 6410 triple quadrupole (QqQ) mass spectrometer. A Poroshell 120 EC-C18 column (150 × 2.1 mm, 2.7 μm, Agilent Technologies) was used for metabolite separation. Targeted LC/MS analysis of PA building blocks and anthocyanins with multiple reaction monitoring (MRM) followed the method established in our previous study (Li et al., 2017).

### 2.5 RNA extraction, library construction, and sequencing

The total RNA of grape suspension cells was isolated using the Universal Plant Total RNA Extraction Kit (BioTeke, Beijing, China). The quality and quantity of the RNA samples were assessed by an Agilent Bioanalyzer 2100 system (Agilent, CA, USA). The cDNA library construction and Illumina Novaseq sequencing were performed by Novogene Biotech Co., Ltd (Beijing, China) following the standard procedures.

### 2.6 Transcript assembly and quantification of gene expression level

To obtain clean reads, raw data were filtered by removing low-quality reads, adapter sequences, and poly-N sequences. The clean reads were then aligned to the *Vitis vinifera* reference genome (version V1) using HISAT2 v2.0.5 with default parameters. Reference-based transcript assembly and novel transcript prediction were performed by StringTie v1.3.3b. Read counts for each gene were obtained by FeatureCounts v1.5.0-p3. Gene expression levels were estimated by calculating the fragments per kilobase of transcript per million mapped reads (FPKM) values. The heatmaps for visualizing gene expression levels were plotted using the Heatmap tool on the Galaxy web-based platform (https://usegalaxy.org/).

### 2.7 qRT-PCR analysis

To validate the RNA-seq results, the same RNA-seq samples were used for qRT-PCR analysis. The RNA was reverse transcribed using HiScript® II Q RT SuperMix for qPCR (+gDNAwiper) (Vazyme, Nanjing, China) following the manufacturer’s instructions. The qRT-PCR reactions were performed using SYBR qPCR Master Mix (Vazyme, Nanjing, China) on an ABI 7300 real-time system (Thermo Fisher, USA). The qRT-PCR program consisted of an initial denaturation at 95°C for 30 s, followed by 40 cycles of denaturation at 95°C for 10 s and annealing/extension at 60°C for 31 s. *VvUbiquitin1* was used as a reference gene to normalize the expression levels of the target genes (Bogs et al., 2005). The relative expression levels were calculated using the 2^-ΔΔCT^ method. The gene-specific primers used in the study are listed in **Supplementary Table 1**.

### 2.8 Differential expression analysis and gene functional enrichment

To identify differentially expressed genes (DEGs) between two experimental groups, the R package DESeq2 1.20.0 was used with the default parameters. The Benjamini and Hochberg method was applied to compute the adjusted *P-*value (padj), which was then used together with the Fold change (FC) to detect significant differences in gene expression. The criteria for significant differential expression were padj < 0.05 and |Log2(FC)| > 1. The DEGs between two groups were visualized by volcano plots using Hiplot (https://hiplot.org), an online platform for interactive data visualization. The DEGs were subjected to GO and KEGG pathway enrichment analysis using TBtools, a software suite for biological data analysis (Chen et al., 2020). The enrichment results were visualized by bubble plots, rose charts, and bee plots using Hiplot. The functionally grouped GO analysis was performed using ClueGO, a Cytoscape plug-in that integrates GO terms and creates functionally organized GO networks (Bindea et al., 2009). The parameters for ClueGO analysis were set as follows: overlap threshold 50%, kappa score 0.5, and the default settings for the other options.

### 2.9 Gene co-expression analysis

Gene co-expression analysis was performed using the R package WGCNA 1.72 with FPKM value matrix as the input. The soft threshold was selected as 6 based on the scale-free topology criterion and the mean connectivity of the network. The minimum number of genes allowed to be included in the module was set to 50. Other parameters for the analysis were kept as the defaults. The co-expression network was visualized by Cytoscape V3.6.1.

### 2.10 Flavonoid related transcription factor annotation

Transcription factor genes in DEGs were identified using BLASTN against *Vitis vinifera* database in Plant Transcription Factor Database (PlantTFDB) V5.0. The identified transcription factor genes were further annotated using BLASTX against a combined database of NCBI nr with the sequences of experimentally characterized flavonoid transcription factors retrieved manually based on the published literatures. NCBI BLAST standalone version 2.13.0+ was used in the above analysis with default parameters.

### 2.11 Statistical analysis

The statistical analysis of the data was conducted using the SPSS software version 21.0. Data were plotted using GraphPad software version 8.0.2.

## 3 Results

### 3.1 75 mM and 150 mM NaCl inhibited the growth of grape suspension cells to different extent

**Figure 1:**
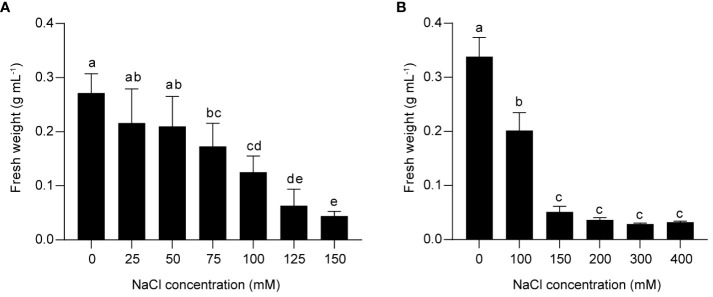
The effects of different NaCl concentrations on the growth of grape suspension cells. The cell suspensions derived from berry skins of *Vitis vinifera* L. Cabernet Sauvignon were treated with various levels of NaCl for two weeks, and the fresh weight was measured as an indicator of cell growth. **(A)** 150 mM NaCl induced a more severe inhibition of cell growth than 75 mM NaCl. **(B)** The growth of grape suspension cells was inhibited by NaCl concentrations above 150 mM, but the fresh weight was not further decreased compared to 150 mM NaCl. Data are presented as the mean ± SD (*n* = 3 biological replicates; different letters above the bars indicate significant differences (P < 0.05) according to one-way ANOVA with Duncan test).

The effect of salinity stress on flavonoid metabolism in grapes was investigated using a suspension cell system derived from berry skins of *Vitis vinifera* L. Cabernet Sauvignon. To determine the optimal salt concentration for inducing stress response, we treated the suspension cells with different concentrations of NaCl for two weeks, and measured the fresh weight as an indicator of cell growth. We found that 75 mM NaCl inhibited cell proliferation, and 150 mM NaCl further reduced cell growth (**Figure 1A**). Higher concentrations of NaCl (200 mM, 300 mM, and 400 mM) did not cause additional decrease in fresh weight compared to 150 mM NaCl (**Figure 1B**). Based on these results, we inferred that 75 mM NaCl was sufficient to induce a significant stress response in grape suspension cells, and that 150 mM NaCl triggered a more severe one. Therefore, it was worth studying the response of PA and anthocyanin biosynthesis under both treatments. For convenience, we used low salinity (L) and high salinity (H) to represent 75 mM and 150 mM of NaCl, respectively.

### 3.2 PA and anthocyanin accumulation in grape suspension cells under NaCl stress

**Figure 2:**
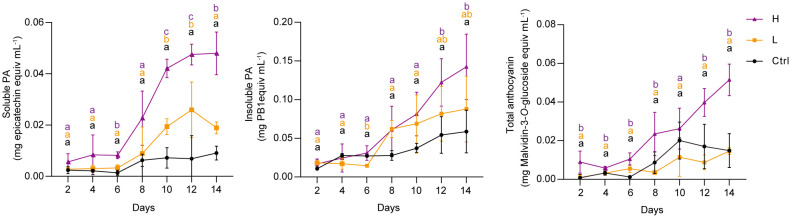
Time series quantification of soluble PAs, insoluble PAs and total anthocyanins in grape suspension cells under 0 mM (Ctrl), 75 mM (L) and 150 mM (H) NaCl treatment conditions. Soluble PAs were extracted using acetone/water solvent followed by chloroform extraction, and insoluble PAs were present in the residues after soluble PAs acetone/water extraction. Soluble PAs were measured by the DMACA method and expressed as epicatechin equivalents. Insoluble PAs were quantified by the butanol-HCl lysis method and expressed as procyanidin B1 (PB1) equivalents. Total anthocyanins were extracted using methanol/water solvent and determined by pH-differential method. The levels of total anthocyanins were expressed as malvidin-3-*O*-glucoside equivalents. Data are shown as the mean ± SD (for *n* = 3 biologically independent samples); the different letters above the bars represent statistically significant differences (*P* < 0.05) determined by one-way ANOVA with Duncan test.

**Figure 3:**
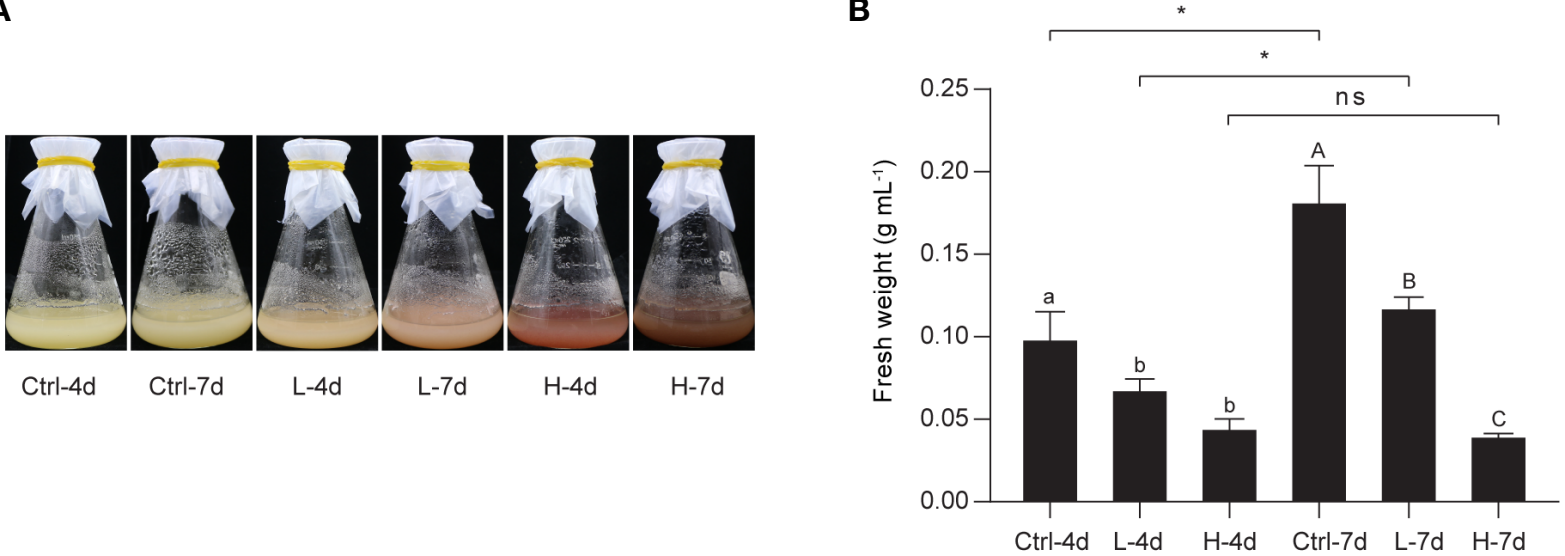
Photographs and fresh weight of the grape suspension cells used for metabolite and transcriptome analysis. **(A)** Photographs of the grape suspension cells treated with 0 mM NaCl (Ctrl), 75 mM NaCl (L) and 150 mM NaCl (H) at day 4 (4d) and day 7 (7d). **(B)** Fresh weight of the grape suspension cells from each treatment group. Data are shown as the mean ± SD (for *n* = 3 biologically independent samples; the different letters above the bars represent statistically significant differences (*P* < 0.05) determined by one-way ANOVA with Duncan test; * *P*< 0.05, two-tailed unpaired Student’s *t* tests; ns, not significant).

To investigate the effect of NaCl stress on PA and anthocyanin biosynthesis in grape suspension cells, we performed time-series assays to monitor the content of these metabolites throughout a culture cycle. We found that soluble PAs and insoluble PAs remained relatively stable under both L and H treatments for the first 6 days, and then increased from day 6 onwards (**Figure 2**). Anthocyanins in H group also accumulated mainly from day 6 to day 14. In contrast, L stress did not induce significant amounts of anthocyanins in suspension cells, similar to the control group (Ctrl) (**Figure 2**). The color of the suspension cells reflected the changes in anthocyanin content. Correspondingly, suspension cells under H at day 4 and day 7 (H-4d and H-7d) showed a deep red color, which was not observed in Ctrl at either day 4 or day 7 (Ctrl-4d and Ctrl-7d) (**Figure 3A**). The cells treated with L for 7 days (L-7d) had a slight pink hue, which was more obvious than that of L samples at day 4 (L-4d) (**Figure 3A**). In addition, L-4d and H-4d samples exhibited growth inhibition phenotype, as indicated by their lower fresh weight than Ctrl-4d (**Figure 3B**). Unlike L treatment group, the fresh weight of H samples did not increase from day 4 to day 7 (**Figure 3B**). These results suggest that the degree of salinity stress experienced by the L and H samples differed, at least from day 4 onwards.

**Figure 4:**
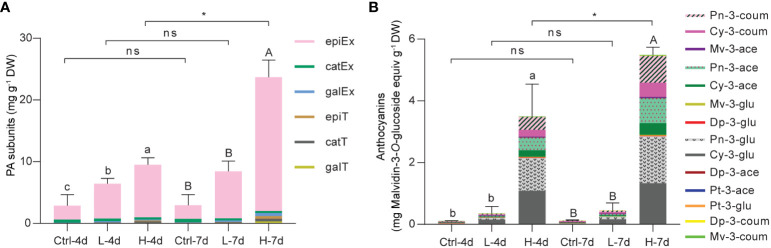
Compositional analysis of PAs and anthocyanins in grape suspension cells under 0 mM (Ctrl), 75 mM (L) and 150 mM (H) NaCl treatment conditions. **(A)** The analysis of composition of PA subunits in each grape suspension cell sample. Dry sample powders were directly subjected to phloroglucinolysis in order to cleavage both soluble and insoluble PAs. The resulting extracts were subjected to LC/MS analysis (epiEx, (-)-epicatechin extension unit; catEx, (+)-catechin extension unit; galEx, (-)-epicatechin-3-*O*-gallate extension unit; epiT, (-)-epicatechin terminal subunit/free monomer; catT, (+)-catechin terminal subunit/free monomer; galT, (-)-epicatechin-3-*O*-gallate terminal subunit/free monomer). **(B)** The analysis of composition of anthocyanins in each grape suspension cell sample. Anthocyanins were extracted with methanol/water and subjected to LC/MS analysis (Pn-3-coum, Peonindin-3-*O*-6”-coumaroyl-glucoside; Cy-3-coum, Cyanidin-3-*O*-6”-coumaroyl-glucoside; Mv-3-ace, Malvidin-3-*O*-6”-acetyl-glucoside; Pn-3-ace, Peonindin-3-*O*-6”-acetyl-glucoside; Cy-3-ace, Cyanidin-3-*O*-6”-acetyl-glucoside; Mv-3-glu, Malvidin-3-*O*-glucoside; Dp-3-glu, Delphindin-3-*O*-glucoside; Pn-3-glu, Peonindin-3-*O*-glucoside; Cy-3-glu, Cyanidin-3-*O* -glucoside; Dp-3-ace, Delphindin-3-*O*-6”-acetyl-glucoside; Pt-3-ace, Petunidin-3-*O*-6”-acetyl-glucoside; Pt-3-glu, Petunidin-3-*O*-glucoside; Dp-3-coum, Delphindin-3-*O*-6”-coumaroyl-glucoside; Mv-3-coum, Malvidin-3-*O*-6”-coumaroyl -glucoside). The content of each type of anthocyanin was expressed as malvidin-3-*O*-glucoside equivalents. Data are shown as the mean ± SD (for n = 3 biologically independent samples; the different letters above the bars represent statistically significant differences (*P* < 0.05) determined by one-way ANOVA with Duncan test; * *P*< 0.05, two-tailed unpaired Student’s *t* tests; ns, not significant).

To gain the insight into the composition of PA building blocks in the current suspension cell system, we directly subjected the ground cell samples to phloroglucinolysis followed by LC/MS analysis. The flavan-3-ol monomers detected by this technique consist of free flavan-3-ols and PA starter units, while phloroglucinol-flavan-3-ol adducts are derived from PA extension units. Quantification results revealed that (-)-epicatechin extension unit was the predominant PA building block in grape suspension cells with or without salinity treatments, while (-)-epigallocatechin-, galloyl-(-)-epicatechin- and (+)-catechin-type subunits together accounted for only one-tenth of the total flavan-3-ols (**Figure 4A**). This suggested that the flux to 2,3-*cis* flavan-3-ols was preferred in the suspension cells, which was the common case in grape berry skins. However, the low levels of (-)-epigallocatechin reflected the fact that C5’-hydroxylation was somewhat blocked in the suspension cell system under NaCl stress. Compared with L treatment, H stress was more effective in inducing total flavan-3-ol accumulation at both day 4 and day 7 (**Figure 4A**). The total flavan-3-ol level of H-7d was twice that of H-4d, while there was no further increase in total flavan-3-ol content in either L or Ctrl groups from day 4 to day 7 (**Figure 4A**).

To examine anthocyanin composition, we subjected 50% methanol/H_2_O (v/v) soluble extracts to LC/MS analysis following the method we developed previously (Li et al., 2017). Consistent with the cell color shown in **Figure 3A**, LC/MS data revealed that anthocyanins were mainly accumulated in H-4d and H-7d samples. The profiles of anthocyanins in these two samples were nearly identical, although total anthocyanin concentration in H-7d was one-third higher than in H-4d (**Figure 4B**). Anthocyanins induced in suspension cells were predominantly cyanidin-based. More than half of these anthocyanins were 3’-*O*-methylated cyanidin-3-*O*-glucoside (also known as peonidin-3-*O*-glucoside), of which over forty percent were further acetylated and coumaroylated (**Figure 4B**). Thus, in despite of a deficiency of 3’5’-hydroxylated anthocyanins, the common types of anthocyanin modifications in grape berry skins existed in the suspension cells under NaCl stress.

### 3.3 NaCl stress-induced gene expression profiles in flavonoid pathway

Transcriptome analysis was performed with Illumina RNA-Seq to investigate the response of gene expression to NaCl stress in the grape suspension cell system. Transcripts were assembled from pair-end reads based on the grape PN40024.V1 genome, and their expression levels were normalized by Fragments Per Kilobase Million (FPKM). Differentially expressed genes (DEGs) between two given conditions were identified using DESeq2 (|Log2(FC)| > 1, padj < 0.05). qPCR data and FRKM values of the selected flavonoid pathway genes correlated well (**Supplementary Figure 1**), suggesting that the RNA-Seq experiment was reliable.

**Figure 5:**
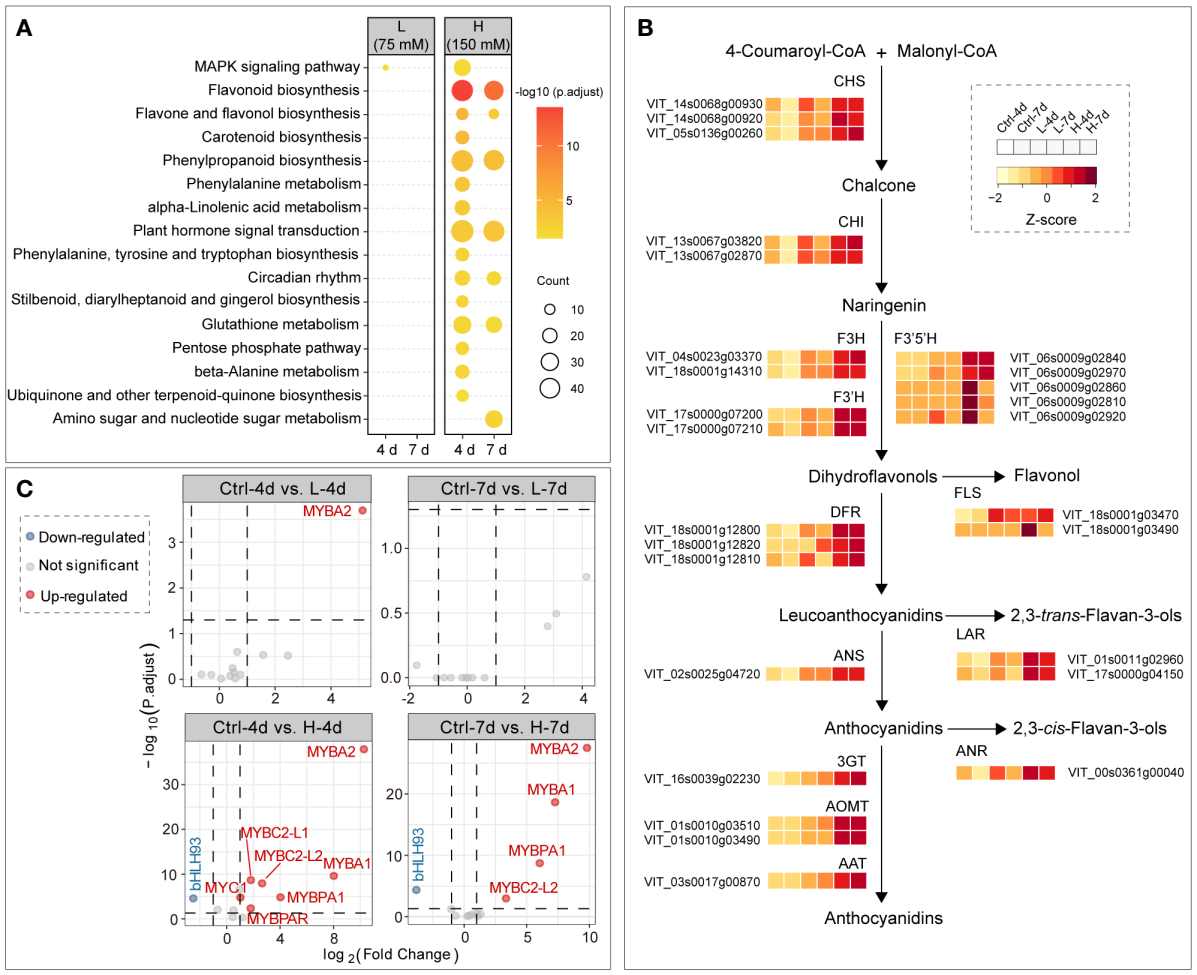
Salinity stress induced gene expression in flavonoid pathway. **(A)** KEGG enrichment of DEGs between salinity treatment and control groups at day 4 and day 7. **(B)** Gene expression patterns in flavonoid pathway among samples. **(C)** Differential expression analysis of well characterized flavonoid transcription factor genes in grape suspension cells, and transcription factor genes *VvMYBA1*/*A2*, *VvMYB5a*/*5b*, *VvMYBC1*, *VvWDR1*, *VvMYBPA1*/*PA2*, *VvMYBPAR*, *VvMYBC2-L1*/*L2*, *VvMYB86* and *VvbHLH93* are included in the plots.

Kyoto Encyclopedia of Genes and Genomes (KEGG) pathway enrichment analysis showed that flavonoid biosynthesis was the most significantly enriched pathway by H treatments, followed by phenylpropanoid biosynthesis and plant hormone signal transduction; DEGs between samples of L and Ctrl groups were not enriched in any KEGG terms, except for a few genes of mitogen-activated protein kinase (MAPK) signaling pathway in L-4d (**Figure 5A**). Correspondingly, the expression levels of the structural genes in flavonoid pathway were generally increased with the increasing NaCl concentration, which was consistent with the salt dose-dependent accumulation of PAs and anthocyanins (**Figure 5B**). Compared with other flavonoid structural genes, the response of *F3’5’H* genes to salinity stress was more complex in the suspension cells. Two of five isoforms of *F3’5’H*s detected by the RNA-Seq assay were mainly expressed in H-4d and H-7d samples, while the transcription of the other three was activated only in H-4d (**Figure 5B**). F3’5’H competes with F3’H for naringenin or dihydrokaempferol to produce 3’5’-hydroxylated backbones of PAs and anthocyanins (Bogs et al., 2006). Similar to the results from a previous study on the expansion and sub-functionalization of *F3’5’H* (Falginella et al., 2010), the unsustainable expression of *F3’5’H* copies was likely responsible for the negligible levels of delphinidin-derived anthocyanins and epigallocatechin-based PAs in the suspension cells.

The PA and anthocyanin biosynthesis related transcription factors, including MYB, bHLH/MYB and WD40/WDR family proteins, have been extensively studied in grapes. However, these works have mostly been carried out in the context of berry development, rather than under stress conditions. VvMYB5a and VvMYB5b activate the general flavonoid pathway in grapevine (Deluc et al., 2006, 2008), but their gene expression levels were not affected by salinity stress in the grape suspension cells (**Figure 5C**). This suggested that additional regulators were required for channeling the flux to flavonoid pathway in grape cells under salinity stress. Two functional isoforms of *MYBA* genes, namely *VvMYBA1* and *VvMYBA2*, are essential for anthocyanin synthesis in red grape cultivars (Walker et al., 2007), and their expression levels were up-regulated for at least 60 times under H treatment, compared to the controls (**Figure 5C**). This corresponded to the high expression levels of anthocyanin genes, including *DFR*, *ANS*, *3GT*, *AOMT* and *AAT*, under H treatment at both day 4 and day 7 (**Figure 5B**). The expression level of *VvMYBPA1*, a gene that activates PA synthesis, increased under H treatment, but not under L treatment. This was in line with the expression patterns of *VvANR* and *VvLAR*s. Whereas the gene encoding VvMYBPA2, another key positive transcription factor in PA pathway (Terrier et al., 2009), was not responsive to the salinity stress, the gene encoding VvMYBPAR, phylogenetically clustered with VvMYBPA2 (Koyama et al., 2014), was up-regulated in H-4d sample. Although two of three MYBC2 PA pathway repressor genes, namely *VvMYBC2-L1* and *VvMYBC2-L2* (Cavallini et al., 2015), were also activated by salinity stress, their inhibition of PA accumulation was not significant in the grape suspension cell system as indicated by the metabolite data. *VvMYBC2* and *VvMYBPA1* are the targets of *VvbHLH93*, which inhibits the expression of *VvLAR*s in PA pathway and promotes the transcription of anthocyanin genes (Cheng et al., 2022). *VvbHLH93* expression level was down-regulated in both H-4d and H-7d samples, which partially explained the similar expression patterns of *VvMYBPA1* and *VvMYBC2* genes under salinity stress (**Figure 5C**). In addition, *VvMYB86*, a gene oppositely regulating PA and anthocyanin biosynthesis (Cheng et al., 2021), was neither responsive to L nor H salinity stress. This was consistent with the similar accumulation patterns of PA and anthocyanin in cells under salt stress conditions.

### 3.4 Identification and co-expression analysis of genes that essentially responded to NaCl stress in grape suspension cells

**Figure 6:**
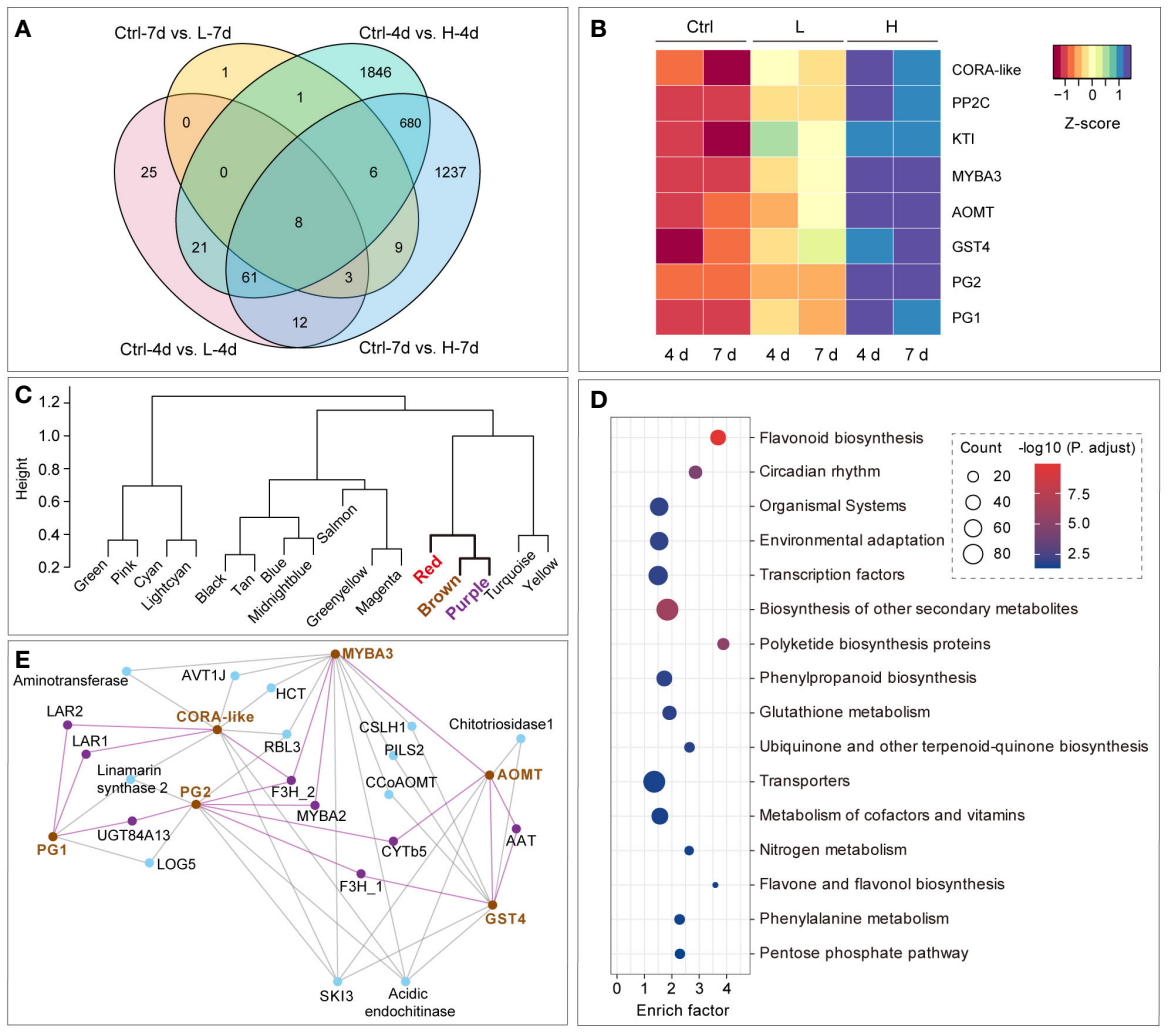
Identification and co-expression analysis of genes consistently involved in NaCl stress response in grape suspension cells. **(A)** Venn diagram of eight genes that were differentially expressed under both high (H) and low (L) salinity at day 4 (4d) and day 7 (7d). **(B)** The expression patterns of the eight genes across all the samples. **(C)** The clustering of module eigengenes based on WGCNA analysis. **(D)** KEGG enrichment of genes in the red, brown and purple modules in **(C)**. **(E)** The co-expression network containing the genes consistently responsive to salinity stress in the brown module. The top 20 co-expression genes for *VvCORA-like*, *VvMYBA3*, *VvAOMT*, *VvGST4* and the two *VvPG*s were retrieved by ranking the weight values from WGCNA analysis, and only the nodes with more than one edge were kept for the network visualization.

We used a Venn diagram to compare the number of DEGs among the four conditions: Ctrl-4d vs. L-4d, Ctrl-7d vs. L-7d, Ctrl-4d vs. H-4d and Ctrl-7d vs. H-7d. The diagram showed that H treatment had a much stronger effect on the transcriptome of the suspension cells than L at both time points (**Figure 6A**). Only eight genes were commonly regulated by both L and H treatments: *VvCORA-like*, *protein phosphatase 2C* (*VvPP2C*), *Kunitz trypsin inhibitor 2* (*VvKTI2*), *VvMYBA3*, *VvAOMT*, *glutathione S-transferase* 4 (*VvGST4*), and two isoforms of polygalacturonases (*VvPG1* and *VvPG2*). These eight genes were all up-regulated by L treatment, and their transcriptions were further enhanced by H treatment (**Figure 6B**). This suggested that they were essential for the response to NaCl stress.

To investigate the potential associations among the eight genes, weighted correlation network analysis (WGCNA) was performed, and 16 modules were identified in total. Among them, *VvCORA-like*, *VvMYBA3*, *VvAOMT*, *VvGST4*, *VvPG1* and *VvPG2* were clustered in the brown module, while *VvPP2C* and *VvKTI2* were assigned to the purple and red modules, respectively (**Supplementary Table 2**). Eigengene-based clustering analysis revealed that the brown, red and purple modules were closely related (**Figure 6C**), indicating that they might share common biological functions. The flavonoid pathway of KEGG term was significantly enriched combining the genes from these three modules (**Figure 6D**). In particular, the majority of flavonoid genes were included in the brown module, while the red and purple modules contained only around 15% of the flavonoid genes, such as a *VvCHI* isoform (VIT_13s0067g03820), a *VvDFR* isoform (VIT_18s0001g12820), *VvANS*, and *VvANR*. This suggested that the brown module was more relevant to salinity-induced anthocyanin and PA biosynthesis.

Within the brown module, VvAOMT catalyzes anthocyanin methylation (Hugueney et al., 2009), and VvGST4 is a PA transporter and the final enzyme to product anthocyanidin (Pérez-Díaz et al., 2016; Eichenberger et al., 2023). Although the SNPs within the *VvMYBA3* locus were tightly associated with grape berry color, the overexpression of *VvMYBA3* in the grape suspension cells did not induce the expression of *Vv3GT* and anthocyanin accumulation (Walker et al., 2007; Fournier-Level et al., 2009; Guo et al., 2019). Moreover, the functions of VvCORA-like, VvPG1 and VvPG2 *in vivo* were still unknown. However, since they co-existed with flavonoid genes in the same module, we speculated that they might have a role in anthocyanin and PA biosynthesis under salinity stress. We retrieved the top 20 co-expression genes for each of these six genes based on the weight values of the adjacency matrix from WGCNA. The gene co-expression network was constructed accordingly, and only the nodes with more than one edge were kept for visualization. The genes in this network were mainly related to flavonoid biosynthesis and cell wall metabolism (**Figure 6E**). The hub genes of this network included the ones encoding shikimate kinase and acidic class III chitinase, which were required for the synthesis of flavonoid precursors and the defense against pathogens and oxidative stress in grapevine, respectively (Almagro et al., 2014; Gonçalves et al., 2019). However, the mechanism of how cell wall composition influences flavonoid biosynthesis under salinity stress was still unclear. Within the network, *VvMYBA3* was directly linked with the well-characterized anthocyanin genes, *VvAOMT*, *VvF3H1* and *VvMYBA2*, as well as a cellulose synthase-like coding gene (*VvCSLH1*) and two lignin synthesis genes, *caffeoyl-coenzyme A O-methyltransferase* (*VvCCoAOMT*) and *hydroxycinnamoyltransferase* (*VvHCT*). *VvAAT* was co-expressed with *VvAMOT* and *VvGST4*, which were also directly connected with each other. *VvPG2* was linked to *VvAMOT* and *VvGST4* through *VvCYTb5* and *VvF3H2*, respectively. *VvLAR1* and *VvLAR2* were both the co-expression partners of *VvPG1*. The two *VvPG*s were also associated with the aid of *VvUGT84A13*, whose homolog in pedunculate oak was identified as a candidate gene involved in gallotannin biosynthesis (Mittasch et al., 2014). CORA-like proteins are believed to play roles in magnesium uptake and storage in plants, and their gene expression is enhanced by stress conditions (Chen and Ma, 2013; Jha et al., 2021). Our data showed that *VvCORA-like* was mainly co-expressed with *VvLAR*s, *VvF3H2*, *VvHCT* and two aminotransferase coding genes, suggesting that it was potentially involved in phenolic metabolism under salinity stress.

### 3.5 Identification of regulator candidates involved in NaCl concentration-dependent PA and anthocyanin accumulations of grape suspension cells

**Figure 7:**
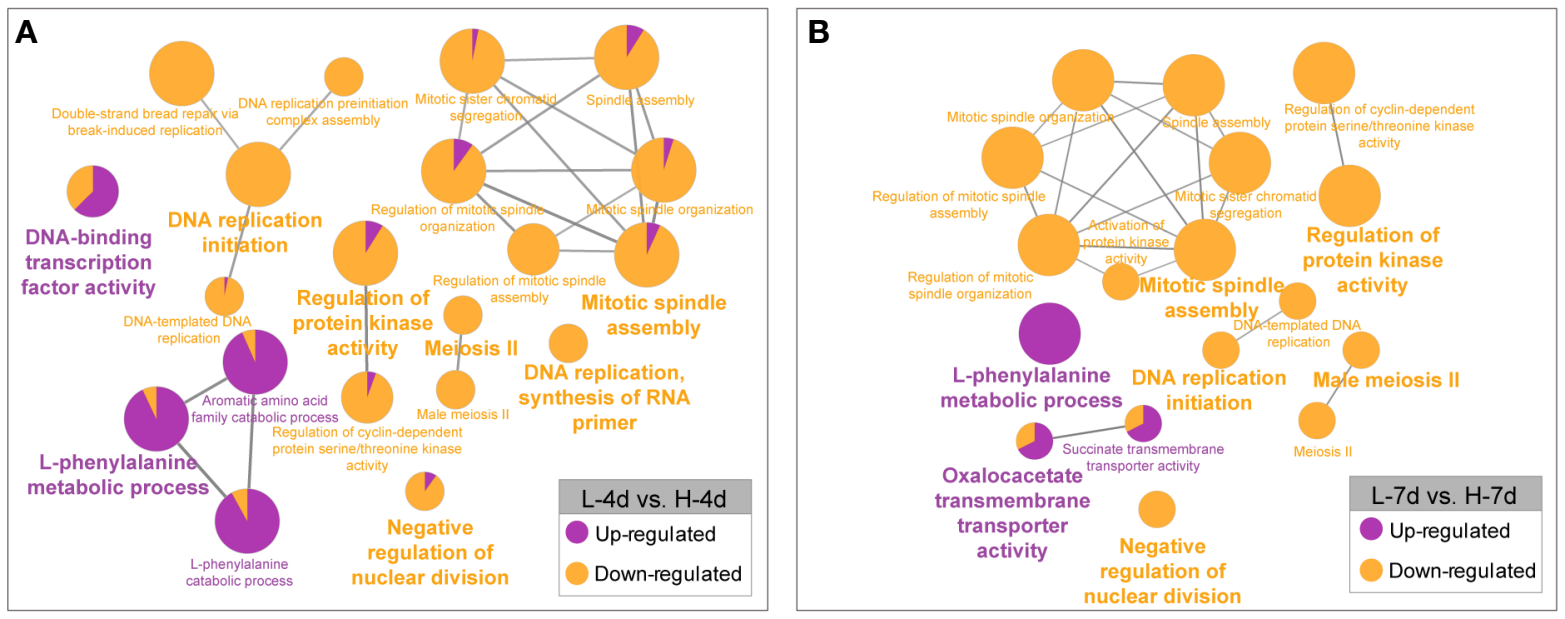
GO enrichment analysis of genes with NaCl dose-dependent expression. The genes that were up- or down-regulated by increasing salinity levels at day 4 **(A)** and day 7 **(B)** were analyzed separately. The results were visualized by functionally grouped networks, which show the relationships among the enriched GO terms. The node size reflects the term enrichment significance, and the proportions of up- and down-regulated genes in each term are shown in purple and orange, respectively.

Besides the several genes that were constitutively responsive to salinity stress, many genes were differentially expressed depending on the NaCl concentration. The GO enrichment analysis revealed that the higher level of NaCl suppressed the expression of genes involved in cell growth processes, such as DNA replication, cell cycle regulation, spindle assembly, RNA primer synthesis, nuclear division inhibition, and meiosis II (**Figure 7**). This further supported the findings that high salt concentrations hindered grape suspension cell proliferation at the transcriptional level. Compared with L treatment, the H treatment enhanced the expression of genes related to phenylalanine and flavonoid metabolism in grape suspension cells at both day 4 and day 7 (**Figure 7**). This suggested the key roles of phenylalanine and flavonoid pathways in coping with the increased salt stress, possibly by enhancing the accumulations of PA and anthocyanin in cells.

To gain insights into the regulators responsible for the NaCl-dose dependent activation of PA and anthocyanin pathways in grape suspension cells, we obtained differentially expressed transcription factor genes (|Log2(FC)| > 1, padj < 0.05) from L-4d vs. H-4d and L-7d vs. H-7d comparison groups, using BLASTN against Plant Transcription Factor Database (PlantTFDB) V5.0. Seventeen transcription factors were further annotated as flavonoid pathway relevant, using BLASTX against a combined database of NCBI nr and experimentally characterized flavonoid transcription factors from crops and model species (**Table 1**). Five flavonoid-relevant transcription factors exhibited NaCl-dose dependent expression only at day 4 but not at day 7: VvMYBC2-L3 (VIT_14s0006g01620), which was down-regulated in H-4d compared with L-4d; and the homologs of *Fragaria* × *ananassa* FAV1 (VIT_11s0037g00010), Arabidopsis NAC32 (VIT_18s0001g02300), *Prunus persica* NAC25 (VIT_00s0375g00040) and VvNAC17 (VIT_19s0014g03290) were up-regulated in H-4d. Among these three NAC transcription factors, AtNAC32 represses the stress-induced anthocyanin synthesis (Mahmood et al., 2016), while PpNAC25 promotes anthocyanin accumulation (Geng et al., 2022). Moreover, Badim et al. (2022) demonstrated that overexpressing VvNAC17 in grape suspension cells induces the biosynthesis of both PA and anthocyanin. FaRAV1 has been shown to activate the general phenylpropanoid and flavonoid pathways, while its homolog in *Malus domestica* (MdRAV1) is found to repress PA biosynthesis (Li et al., 2020; Zhang et al., 2020). At both day 4 and day 7, the expression of VIT_01s0011g03070 (a homolog of *MdRVA1*), VIT_18s0001g09250 (a homolog of the anthocyanin pathway repressor *AtLBD38*) (Rubin et al., 2009), and VIT_08s0058g01390 (a flavonoid pathway repressor, *VvWRKY70*) were down-regulated in the H samples compared with the L groups. The expression levels of the homologs of anthocyanin pathway activators *OsMYB3* from rice (VIT_04s0008g01800) (Zheng et al., 2021) and *MdERF109* from apple (VIT_03s0063g00460) (Ma et al., 2021), the classical flavonoid activators *VvMYBPA1* and *VvMYBA1*/*2*/*3*, and that of the well characterized PA negative regulators *VvMYBC2-L1* and *VvMYBC2-L2* were increased with the elevation of NaCl concentration at both time points. The up-regulation of *VvMYBC2-L1* expression may partially associate with the down-regulation of *VvMYBC2-L3* transcript level, as VvMYBC2-L3 is a *VvMYBC2-L1* negative regulator in grapevine (Cavallini et al., 2015). This indicates that VvMYBC2-L family proteins can fine-tune each other to regulate flavonoid biosynthesis under salinity stress. A homolog of Arabidopsis *AtERF4* (VIT_19s0014g02240), which is proposed to promote light-induced anthocyanin biosynthesis (Koyama and Sato, 2018), did not exhibit NaCl-dose-dependent expression at day 4 but showed lower expression level at day 7 as NaCl stress increased. These findings extended our understanding of the coordination of various of flavonoid/phenepropanoid activators and suppressors to cope with different levels of salinity stress.

**Table 1:** List of the annotated flavonoid transcription factor genes with NaCl dose-dependent expression.

H-4d/L-4d Log2 Ratio	H-7d/L-7d Log2 Ratio	*Vitis vinifera* Transcript ID	Best Match	Species	Characterized Function	References
2.31	–	VIT_11s0037g00010	FaRAV1	*Fragaria × ananassa*	Activating phenylpropanoid and flavonoid biosynthesis	(Zhang et al., 2020)
-1.10	–	VIT_14s0006g01620	VvMYBC2-L3	*Vitis vinifera*	Inhibiting PA accumulation	(Cavallini et al., 2015)
1.05	–	VIT_18s0001g02300	AtNAC32	*Arabidopsis thaliana*	Repressing anthocyanin biosynthesis in response to sucrose treatment, high light and oxidative stress	(Mahmood et al., 2016)
1.32	–	VIT_00s0375g00040	PpNAC25	*Prunus persica*	Promoting anthocyanin biosynthesis	(Geng et al., 2022)
1.38	–	VIT_19s0014g03290	VvNAC17	*Vitis vinifera*	Inducing the synthesis of flavonoid	(Badim et al., 2022)
-1.91	-3.25	VIT_01s0011g03070	MdRAV1	*Malus domestica*	Repressing PA biosynthesis	(Li et al., 2020)
5.92	4.88	VIT_03s0063g00460	MdERF109	*Malus domestica*	Promoting light-induced anthocyanin biosynthesis	(Ma et al., 2021)
-4.51	-5.09	VIT_18s0001g09250	AtLBD38	*Arabidopsis thaliana*	Repressing anthocyanin biosynthesis	(Rubin et al., 2009)
-2.80	-2.74	VIT_08s0058g01390	VvWRKY70	*Vitis vinifera*	Inhibiting flavonoid biosynthesis	(Wei et al., 2023)
1.23	3.48	VIT_04s0008g01800	OsMYB3	*Oryza sativa*	Promoting anthocyanin biosynthesis	(Zheng et al., 2021)
1.28	1.32	VIT_01s0011g04760	VvMYBC2-L1	*Vitis vinifera*	Inhibiting PA accumulation	(Huang et al., 2014)
2.51	3.76	VIT_17s0000g02660	VvMYBC2-L2	*Vitis vinifera*	Repressing anthocyanin biosynthesis	(Zhu et al., 2019)
2.46	2.95	VIT_15s0046g00170	VvMYBPA1	*Vitis vinifera*	Promoting PA biosynthesis	(Bogs et al., 2007)
5.58	4.49	VIT_02s0033g00410	VvMYBA1	*Vitis vinifera*	Promoting anthocyanin biosynthesis	(Walker et al., 2007)
5.12	5.67	VIT_02s0033g00390	VvMYBA2	*Vitis vinifera*	Promoting anthocyanin biosynthesis	(Walker et al., 2007)
5.89	5.20	VIT_02s0033g00450	VvMYBA3	*Vitis vinifera*	Possibly promoting anthocyanin biosynthesis	(Guo et al., 2019)
–	-2.50	VIT_19s0014g02240	AtERF4	*Arabidopsis thaliana*	Promoting light-induced anthocyanin biosynthesis	(Koyama and Sato, 2018)

The transcription factors were annotated by using BLASTX against a combined database of NCBI nr and experimentally characterized flavonoid transcription factors from crops and model species. The best match indicates the most similar transcription factor from another plant species that has a characterized function in flavonoid biosynthesis. The species column shows the scientific name of the plant species from which the best match was derived.

### 3.6 Salinity stress duration-dependent differential gene expression in the grape suspension cells

**Figure 8:**
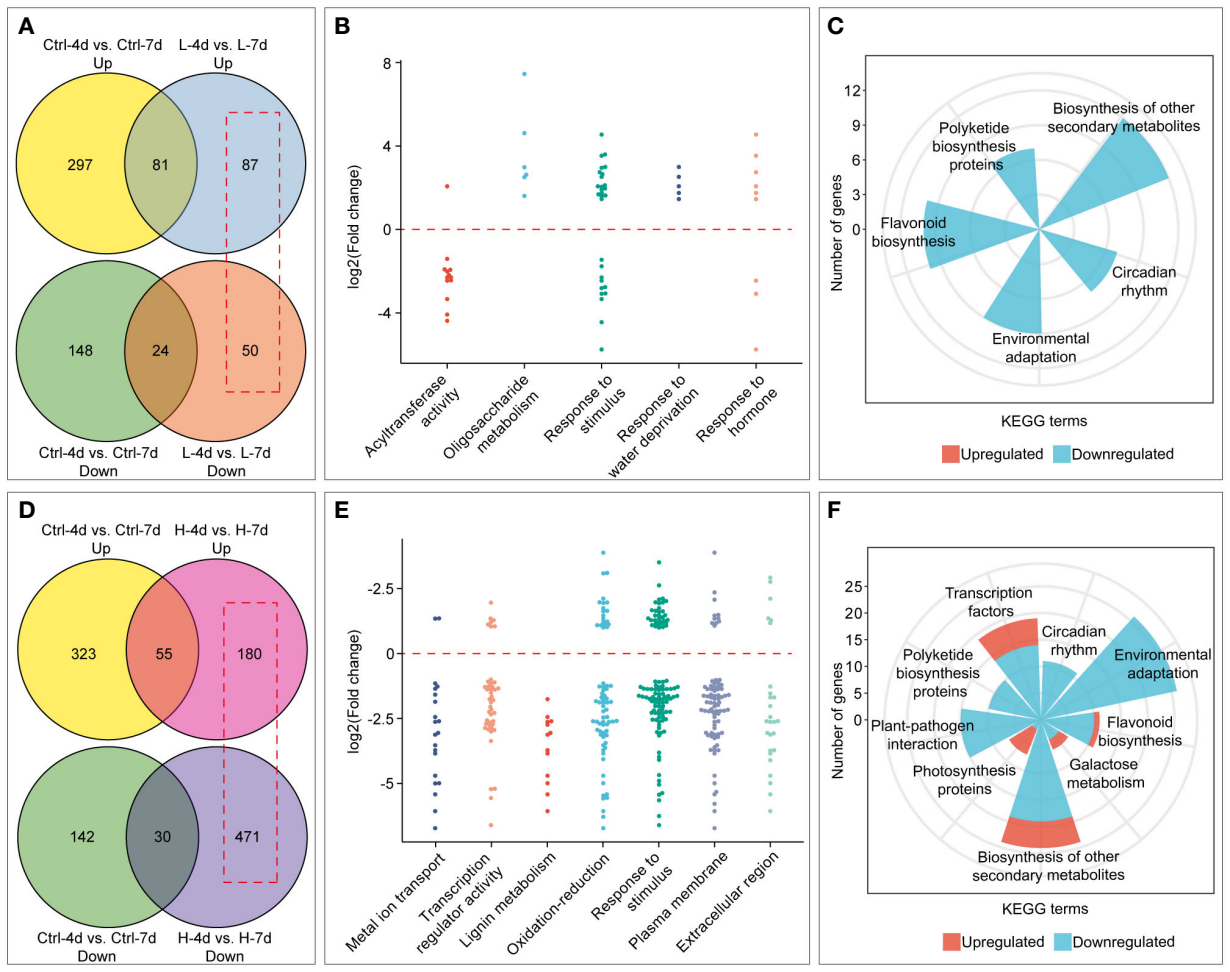
Identification and enrichment analysis of genes with time-dependent expression under low (L) and high (H) salinity stress. **(A)** and **(D)** Venn diagrams showing the number of genes with time-dependent expression under low and high salinity conditions, respectively, outlined in red boxes. **(B)** and **(E)** GO enrichment analysis of genes with time-dependent expression under low and high salinity conditions. Each dot represents the Log2(FC) by comparison between day 7 and day 4. **(C)** and **(F)** KEGG enrichment results of genes with time-dependent expression under low and high salinity conditions.

Salinity stress duration also had varying effects on PA content, anthocyanin accumulation, and cell fresh weight in the H and L groups (**Figures 3A**, **4**). We identified salinity stress duration-dependent DEGs from the RNA-Seq data by excluding the DEGs that were only affected by cultivation time in the control groups (**Figures 8A, D**). The Venn diagram analysis showed that the L group had fewer DEGs than the H group with increasing salinity stress duration. The GO enrichment analysis showed that the L group DEGs were mainly enriched in biological processes or molecular functions related to acyltransferase activity, oligosaccharide metabolism, response to stimulus, water deprivation, and hormone (**Figure 8B**). The KEGG enrichment analysis showed that the L group DEGs had down-regulated expression of genes involved in flavonoid, polyketide and other secondary metabolite biosynthesis, circadian rhythm, and environmental adaptation pathways (**Figure 8C**). This at least explained why the extended L treatment did not result in an increase in PA and anthocyanin.

**Figure 9:**
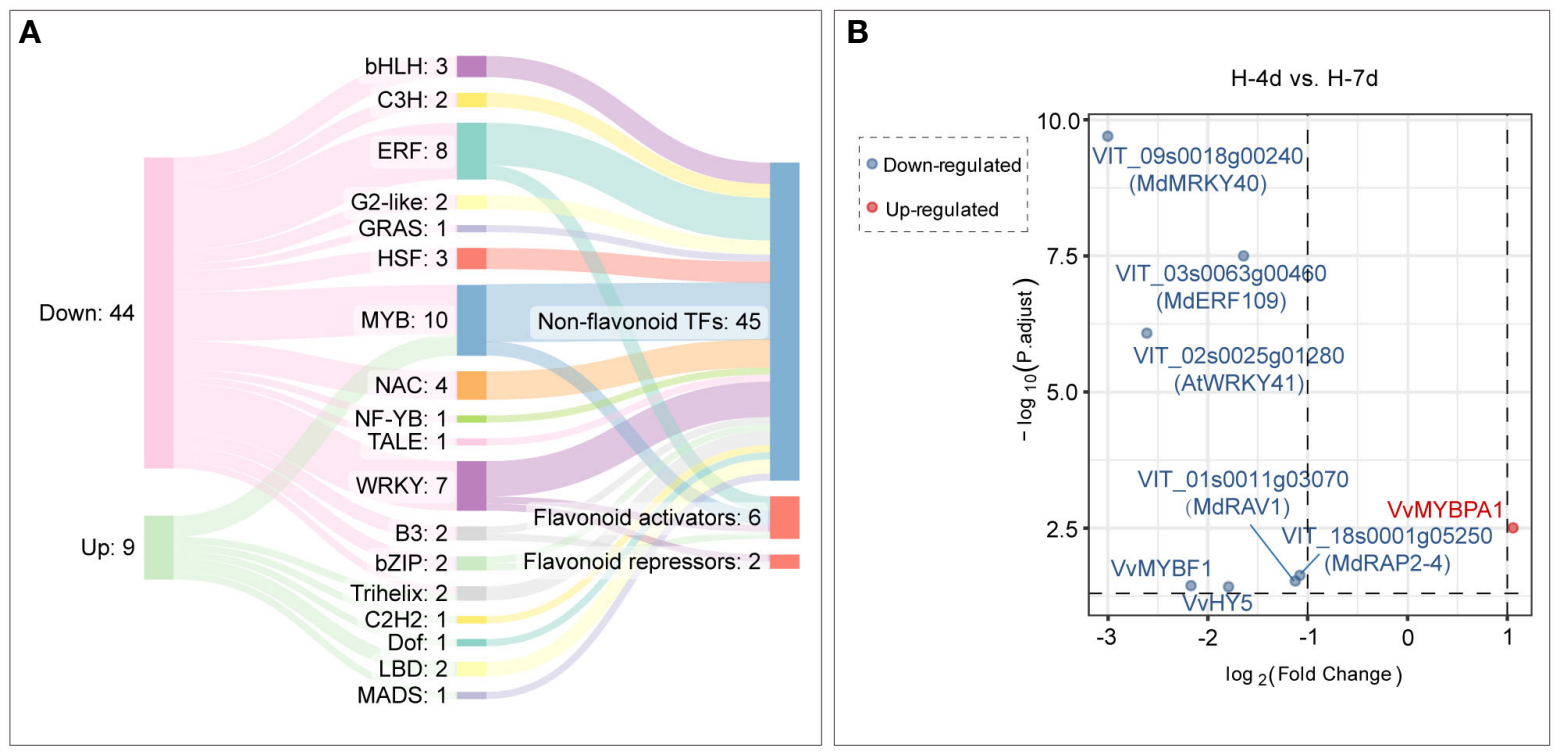
Transcription factor genes with time-dependent expression under high salinity stress. **(A)** A Sankey plot showing the expression changes, protein families, and functional annotations of transcription factor genes under high salinity stress. The left column indicates the gene expression changes, the middle column indicates the protein families that the genes encode, and the right column indicates the functional annotations of the genes. **(B)** Differential expression analysis of flavonoid transcription factor genes in response to durations of high salinity treatment in grape suspension cells.

Compared with H-4d samples, the H-7d samples showed significant changes in gene expression patterns in grape suspension cells. A total of 651 differentially expressed genes (DEGs) were identified, of which 180 were up-regulated and 471 were down-regulated (**Figure 8D**). The GO enrichment analysis revealed that the extended H treatment duration may affect several biological processes or molecular functions, such as metal ion transport, lignin metabolism, response to stimulus, and oxidation-reduction (**Figure 8E**). These processes were likely related to the altered gene expression levels of genes associated with plasma membrane and extracellular region (**Figure 8E**). Moreover, KEGG pathway enrichment showed that these genes mainly involved in plant-pathogen interaction, environmental adaptation, polyketide biosynthesis, circadian rhythm, flavonoid biosynthesis, photosynthesis pathway, galactose metabolism and other secondary metabolism pathways (**Figure 8F**). Although flavonoid pathway was generally less active in H-7d samples compared with that of H-4d, the levels of PAs and anthocyanins were still increased as the salinity treatment prolonged (**Figure 4B**). This phenomenon might be partially attributed to the up-regulation of genes within the flavonoid pathway, as well as the enhanced transcription of genes related to photosynthesis, which produces precursors for flavonoids (Shao et al., 2022). In addition to the biological processes and pathways mentioned above, both GO and KEGG analyses suggested that transcription factors (TFs) were also enriched in the DEGs whose expression were affected by the duration of H stress (**Figure 8E**). Annotated by PlantTFDB database, there were a total of 53 TF genes (belonged to 18 protein families) differentially expressed as the H treatment time extended, with 44 being down-regulated and 9 being up-regulated (**Figure 9A**). Among these TFs, the proteins from ERF, MYB and WRKY families accounted for nearly a half. According to the biochemical evidence and bioinformatic prediction, 6 and 2 TF genes were classified into flavonoid activators and repressors, respectively (**Figure 9A**). Compared with the H-4d, the expression level of PA pathway specific activator *VvMYBPA1* was increased by one-fold in the H-7d sample, while the expression of other proposed PA or anthocyanin activators, including *VvHY5*, *VvMYBF1*, and the homologs of *MdWRKY40*, *MdERF109* and *MdRAP2-4* (VIT_09s0018g00240, VIT_03s0063g00460, and VIT_18s0001g05250) (Shin et al., 2013; An et al., 2019; Li et al., 2020; Wang et al., 2020; Ma et al., 2021) was down-regulated from day 4 to day 7 under H stress (**Figure 9B**). This means that VvMYBPA1 is crucial for maintaining the PA accumulation as H treatment time extended, even though the general flavonoid pathway was less active. Moreover, the expression levels of the proposed flavonoid repressor VIT_01s0011g03070 and VIT_02s0025g01280 (homologs of *MdRAV1* and *AtWRKY41*, respectively) (Ding et al., 2014; Li et al., 2020) were lower in H-7d sample than in H-4d, indicating that they might also contribute to the increased level of anthocyanin, from day 4 to day 7 under H stress.

## 4 Discussion

Plant cell suspensions offer a convenient system to study the response of secondary metabolism to stress stimuli *in vivo* (Onofrio et al., 2009). In this study, we used the grape berry skin-derived suspension cells to investigate the response of PA and anthocyanin metabolism to salt stress. Under both low and high levels of NaCl stress, the grape suspension cell system was able to accumulate PA and anthocyanin, to different extents. PAs and anthocyanin pathways share the common precursor leucoanthocyanidins, whose number of hydroxyl groups on the B-ring is determined by the upstream F3’H and F3’5’H. Although the levels of the five isoforms of VvF3’5’H transcripts were up-regulated by high levels of NaCl stress, the 3’5’-OH PA building blocks and anthocyanins were deficient in the present suspension cell system, which is not the case in Cabernet Sauvignon berry skins. There are more than 10 copies of VvF3’5’Hs present in the grape genome, and the variant 3’5’-OH anthocyanin profiles are associated with the pool of *VvF3’5’H* transcripts across different cultivars (Falginella et al., 2010). Considering this, the protein products of the detected *VvF3’5’H* transcripts were not catalytically efficient enough to compete for substrates with DFR and FLS. A previous study in Arabidopsis showed that distinct sets of anthocyanins can be induced by different abiotic stress conditions, and suggested that the types of decoration of anthocyanin may be used for unique purposes for stress response (Kovinich et al., 2014). Then it is possible that 3’5’-OH decoration of flavonoid backbone was not essentially required for solely responding to salt stress in grapevine, while the abundant levels of delphinidin-based anthocyanin and epi(gallo)catechin-based PAs may at least results from the interaction between temperature and light conditions in the grape berries grown in the field (Azuma et al., 2012).

Among the well characterized flavonoid regulators, *VvMYB5a* and *VvMYB5b* have been found to encode key transcription factors to activate the general flavonoid pathway in grapes (Deluc et al., 2006, 2008; Cavallini et al., 2014). However, neither of these two genes was responsive to salinity stress in the grape suspension cells. This suggests that the expression of *VvMYB5a* and *VvMYB5b* is not induced by salt stress, but might be more relevant to other environmental stimuli, e.g., light (Matus et al., 2009). VvMYBPA1 and VvMYBPA2 are the key PA specific activators in grape seeds and skins, respectively (Bogs et al., 2007; Terrier et al., 2009), while only *VvMYBPA1* but not *VvMYBPA2* was induced by the salt stress in grape suspension cells, which were derived from grape skins in the present study. This means that the salt stress-induced flavonoid regulation network does not essentially present in a tissue-specific manner in grape suspension cells. Moreover, *VvMYBPA1* expression was enhanced by the increased salt stress level and the extended treatment time of high salinity. The genetic evidence showed that VvMYBPA1 not only activates PA branch genes, including *VvLAR1* and *VvANR*, but also positively regulates the expression of *VvCHI* and *VvF3’H* at the upstream of flavonoid pathway (Terrier et al., 2009). Consistent with the similar expression pattern as *VvMYBPA1* in response to the high salt stress, *VvMYBA1*, *VvMYBA2* and *VvNAC17* also positively target to the general flavonoid pathway (Badim et al., 2022) - this might be a strategy to compensate the absence of the functions of VvMYB5a and VvMYB5b in grape suspension cells under salinity stress. Moreover, based on homology, we identified a number of potential flavonoid transcription factors whose expressions were NaCl dose-dependent in grape suspension cells, including MYB, RAV, NAC, LBD, ERF and WRKY family proteins. Most of these transcriptional factor genes were not differentially expressed as the high salinity treatment time extended, suggesting that the regulation network they participated in might be essential for maintaining the flux channeling to PAs and anthocyanins over the time.

The expression levels of genes involved in stress response were generally decreased as the treatment duration extended, under both high and low levels of salinity stress, as shown by GO and KEGG analysis. This might indicate that grape cells were able to alleviate salinity stress over time, which was in line with the notion that PAs and anthocyanins could help plants with stress adaptation (Li and Ahammed, 2023; Yu et al., 2023). The high salinity stress also inhibited cell proliferation, resulting in no change in cell number between day 4 and day 7, whereas the low salinity stress did not severely affect cell growth, leading to an increase in cell number over time. Genetic evidence showed that high levels of PAs and anthocyanin might be cytotoxic and thus result in the high rate of the cell death (Sharma and Dixon, 2005). Recently, VvbHLH93 was identified as a flavonoid pathway suppressor in the grapevine, and its proposed biological significance is to prevent the over-accumulation of phenolic compounds that may negatively affect berry development (Cheng et al., 2022). The present study showed that *VvbHLH93* expression was specifically down-regulated by the high salinity stress and remained at the same level between day 4 and day 7 in the suspension cells. Although the two PA pathway suppressors VvMYBC2-L1 and -L2 (Huang et al., 2014) were up-regulated by the salinity stress, their inhibition effects on flavonoid pathway were negligible as indicated by the metabolite data. This raises the question of whether there are any regulators can potentially balance the salinity stress response and the flavonoid production. One candidate is *VvERF109*, which was up-regulated by the increased level of salinity stress but down-regulated by the extended stress duration in grape suspension cells. ERF109 homologs have diverse roles: they promote anthocyanin accumulation in apple by targeting CHS, UFGT, and bHLH family regulators (Ma et al., 2021); regulate salt stress by influencing programmed cell death, ROS scavenging product biosynthesis (including phenylalanine), and hormone signal transduction in Arabidopsis (Khandelwal et al., 2008; Bahieldin et al., 2016, 2018); and respond to Noble rot infection in grape berries (Pogány et al., 2022). These studies suggest that VvERF109 might have a dual function in maintaining redox homeostasis and fine-tuning the flavonoid content to cope with the stress in grape suspension cells.

As indicated by WGCNA analysis, six out of the eight genes that consistently responded to both low and high salinity stress were present in the same module, including *VvGST4*, *VvAOMT*, *VvMYBA3*, *VvCORA-like*, and two polygalacturonase genes. Co-expression network construction showed that these six genes were associated with each other through the genes related to flavonoid and cell wall compositions. However, exactly how flavonoid pathway coordinated with the cell wall remodeling under salinity stress remains to be elucidated. The function of VvGST4 has been recently clarified to convert intermediate products of ANS into anthocyanidins (Eichenberger et al., 2023), and VvAOMT could subsequently catalyze the methylation of anthocyanins (Hugueney et al., 2009). In addition, the expression of anthocyanin activator *VvMYBA2* was already induced under low salinity stress, while only at higher salt concentrations did the expression levels of PA pathway related transcription factor genes differ from the control groups. Taken together, the above evidence suggests that anthocyanin pathway is more sensitive than PA branch in response to the salinity stress in grape suspension cells. From the perspective of application, besides being the targets for enhancing salt tolerance of crops (Mansour, 2023), *VvGST4* and *VvAOMT* might also be the potential molecular indicators of salt stress in grape cells.

## Data availability statement

The datasets presented in this study can be found in online repositories. The names of the repository/repositories and accession number(s) can be found below: NCBI Sequence Read Archive, PRJNA1048521.

## Author contributions

KZ: Conceptualization, Data curation, Investigation, Methodology, Writing – original draft. YL: Investigation, Methodology, Writing – review & editing. YS: Conceptualization, Writing – review & editing. CD: Conceptualization, Funding acquisition, Resources, Writing – review & editing. KY: Conceptualization, Funding acquisition, Project administration, Resources, Writing – review & editing, Writing – original draft.
