# Supplementary material for: Metabolite and transcriptome analyses reveal the effects of salinity stress on the biosynthesis of proanthocyanidins and anthocyanins in grape suspension cells
Source: Front Plant Sci. 2024 Mar 21;15:1351008. doi: 10.3389/fpls.2024.1351008 (PMC10993317; doi:10.3389/fpls.2024.1351008)
Supplement: Supplementary file 2 [file Presentation_1.pdf]

Supplementary Material

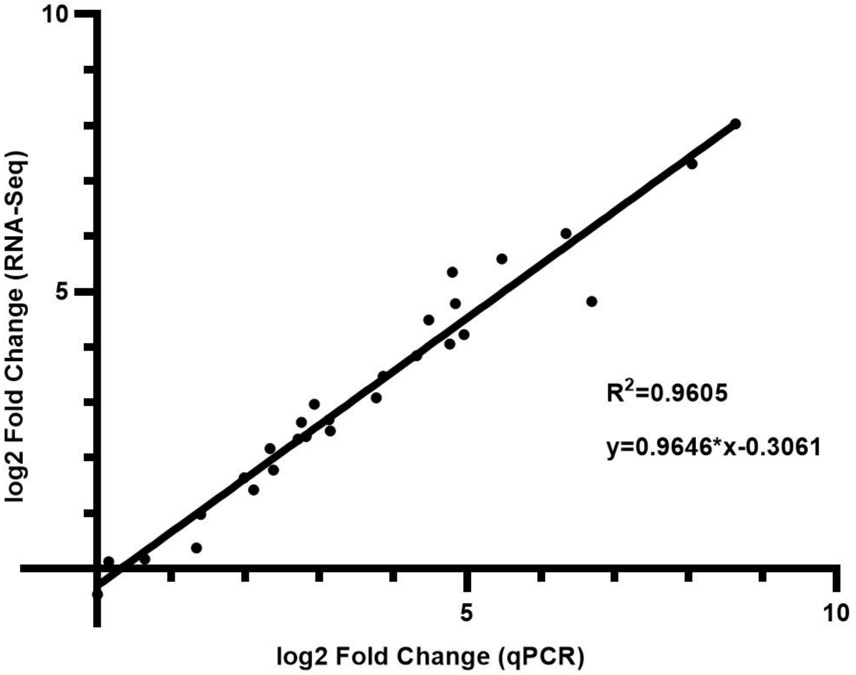

Supplementary Figure 1. Correlation of log2(fold change) values obtained from RNA-seq and qPCR for target genes. The fold change values represent the gene expression ratio of either high (H) to low (L) stress or low (L) to control (Ctrl) stress at different time points. The target genes are *VvMYBA1*, *VvANR*, *VvCHI*, *VvMYBF1*, *VvFLS*, *VvLAR1*, and *VvMYB5*.

Supplementary Table 1. Sequences of primers used in this work

Gene	Primer names	Sequence (5’-3’)
VvMYBA1 (VIT_02s0033g00410)	MYBA1-F	ACTATTGGCATAGTCACCACTTC
	MYBA1-R	GGCAAGGCTTTGGAGAACTTG
VvANR (VIT_00s0361g00040)	ANR-F	GCTGCTGTTACCATCAATCA
	ANR-R	GCAGGATAGCCCCAAGTAGG
VvCHI (VIT_13s0067g03820)	CHI-F	CAGGCAACTCCATTCTTTTC
	CHI-R	TTCTCTATCACTGCATTCCC
VvMYBF1 (VIT_07s0005g01210)	MYBF1-F	GGAGGTTGAGGGGTTGTG
	MYBF1-R	AAGTTGGGGAAGAGCAGGAG
VvFLS (VIT_18s0001g03470)	FLS-F	GCTGCGAACGAGGAGTATGC
	FLS-R	AACGTACCCTCTCCCAGACC
VvLAR1 (VIT_01s0011g02960)	LAR1-F	AAATGAACTCGCATCTGTGT
	LAR1-R	CTGTGGGATGATGTTTTCTC
VvMYB5 (VIT_15s0046g00170)	MYB5-F	TTGACGGGGTTGACTTCTTC
	MYB5-R	GAGTAGTGATTCGGCGAAGG
VvUbiquitin1 (VIT_16s0098g01190)	UBQ-F	GTGGTATTATTGAGCCATCCTT
	UBQ-R	AACCTCCAATCCAGTCATCTAC
